# Community treatment orders and care planning: How is engagement and decision‐making enacted?

**DOI:** 10.1111/hex.13329

**Published:** 2021-08-12

**Authors:** Suzanne Dawson, Eimear Muir‐Cochrane, Alan Simpson, Sharon Lawn

**Affiliations:** ^1^ School of Allied Health Science and Practice, Faculty of Health and Medical Sciences University of Adelaide Adelaide Australia; ^2^ College of Nursing and Health Sciences Flinders University Adelaide Australia; ^3^ Mental Health Directorate Central Adelaide Local Health Network Adelaide Australia; ^4^ Health Service and Population Research Department, Florence Nightingale Faculty of Nursing, Midwifery and Palliative Care, King's College London Institute of Psychiatry, Psychology & Neuroscience London UK; ^5^ College of Medicine and Public Health Flinders University Adelaide Australia

**Keywords:** care planning, community treatment orders, decision‐making, trust

## Abstract

**Background:**

In many jurisdictions worldwide, individuals with a mental illness may be forced to receive care and treatment in the community. In Australia, legislation states that such care should be driven by a care plan that is recovery‐focussed. Key components in the care planning process include engagement and decision‐making about a person's support needs and care options, with trust being an essential component of care planning relationships.

**Objective:**

This study examines how these components were enacted during service care contacts for individuals on community treatment orders.

**Methods:**

The study was located at two community mental health teams in South Australia. Ethnographic observations of care planning discussions between consumers, their carers and clinicians, and interviews with individuals from these groups, were conducted over 18 months. Carspecken's critical ethnography provided a rigorous means for examining the data to identify underlying cultural themes that were informing day‐to‐day care interactions.

**Results:**

Care planning was not occurring as it was intended, with service culture and structures impeding the development of trusting relationships. Clinicians striving to work collaboratively with consumers had to navigate a service bias and culture that emphasized a hierarchy of ‘knowing’, with consumers assumed to have less knowledge than clinicians.

**Conclusions:**

Services and clinicians can challenge prejudicial ethical injustice and counter this through testimonial justice and implementation of tools and approaches that support genuine shared decision‐making.

**Patient or Public Contribution:**

This study included individuals with lived experience of mental illness, their carers and clinicians as participants and researchers.

## 1 BACKGROUND

Care planning in mental health services guides the delivery of care that aims to meet individuals' personalized needs.^2^ Care planning relationships have been found to be central to consumers' experiences of services,^3^ with trust considered an essential component of effective therapeutic relationships.^4^ Epistemic trust, or an individual's willingness to consider knowledge provided by another as trustworthy and relevant,^5^ is essential in the context of care planning relationships, which are promoted as active partnerships within recovery frameworks.^6^, ^7^ In the context of mental health care, there are significant challenges that exist for consumers with regard to the development of trust, particularly for consumers who are subject to legally forced treatment. Community treatment orders (CTOs) are legislated in more than 75 jurisdictions worldwide, and require individuals to comply with treatment plans for their mental illness whilst residing in the community.^8^, ^9^ This paper reports on an ethnographic study conducted in South Australia that explored care planning with consumers on CTOs, with a focus on engagement and decision‐making.

Trust is a communicative action between individuals that is context‐specific and fundamental to effective community living and health care.^4^, ^10^, ^11^ Trust can be conceptualized as an alliance based on the belief that the trustee's best interests will be maintained by the trusted.^12^ Conversely, mistrust is related to a perceived lack of control and agendas that do not align.^4^ The heightened importance and necessity of trust are emphasized in mental health care due to the vulnerability of individuals seeking care from services.^4^ Coercive practices in mental health care, however, are common,^13^ with forced treatment consistently raised by consumers as a primary barrier to the provision of care that is supportive of positive relationships and recovery.^14^, ^15^ Consumers who are already vulnerable experience a further loss of control and power in decision‐making about their own care.^15^

Theories on trust place differing emphases on the roles of personal relationships and social systems in the formation of trust, with Giddens^16^, ^17^ emphasizing the importance of interpersonal relationships and Luhmann^18^, ^19^ emphasizing the role of systems.^20^ Both theories are relevant to care planning for consumers on CTOs. CTOs can create distrust in the health care system and the clinicians and consumers within the system.^21^, ^22^ There are significant and valid justifications for consumers to have reduced trust at the interpersonal level (with clinicians) and the systems level (with services). Such factors include the iatrogenic effects of treatment (medication side effects and coercion),^10^ common features that people with a serious mental illness experience (such as paranoia)^4^ and trauma experienced before and from care contacts.^19^, ^23^, ^24^ In the context of forced treatment, consumers may not believe that clinicians or services are prioritizing their best interests.

Care planning should be collaborative, personalized and recovery‐focused,^6^ however, shared decision‐making (SDM); which promotes autonomy and collaboration, continues to have limited uptake in mental health services.^25^ Decision‐making regarding CTO use is based on clinician perceptions of consumers' level of risk, insight and perceived lack of capacity and engagement with services, specifically, medication compliance.^26^, ^27^, ^28^ While CTOs are considered a treatment tool for enhancing consumer stability and safety, they are also used to reduce risk and prevent harm.^29^, ^30^ Although recent studies have explored decision‐making regarding reasons for CTO use, explorations of ongoing involvement in decision‐making during all care contacts whilst the person is subject to a CTO have been neglected.

Conflicts in decision‐making processes exist for all participants in the care planning relationship. Consumers and carers persistently report lack of involvement in decision‐making in mental health care.^31^ Additionally, workers have also described differing levels of involvement and consequences in decision‐making processes, with psychiatrists feeling exposed to blame for enacting forced care.^32^ Despite evident conflicts for all, consumers on CTOs are the most compromised due to the inherent power differentials between them and clinicians tasked with imposing forced treatment. While some clinicians are sensitive to the tension between a person's autonomy and experience of coercion, studies report that the majority of clinicians do not ask consumers directly about this, nor consider that CTOs are coercive beyond enforcement of medication.^27^, ^29^ Research, however, continues to highlight the significant and negative impacts of coercion that consumers experience being on a CTO.^33^, ^34^

International criticism of public mental health care services emphasizes shortfalls and barriers to provision of recovery‐oriented care, with forced care and care planning being key issues in these critiques.^35^ The ethnographic study reported here found that consumers on CTOs were often positioned by the clinical team as ‘risky’ and ‘insight(less)’, which negatively biased clinicians who were attempting to develop alliances with individuals.^36^ This paper focuses on how everyday care planning discussions between mental health clinicians and consumers impacted on the development of trust. Given the value of and complexity for all parties of negotiating trust in the context of CTO use, an in‐depth examination of care planning dialogues as they occur in day‐to‐day practice was conducted to elucidate barriers and facilitators to the development of trust and thereby care provision that is recovery‐focused in this context.

## 2 METHODS

Carspecken's^1^ critical ethnography was the methodology applied for this study. Ethics approval was granted from the local health network and university human ethics committees (HREC/16/RAH/148). The study site was two community mental health teams in Adelaide, South Australia. Data were collected between June 2017 and December 2018. Information about the project was presented by the first author to the clinical teams. Clinicians approached consumers on CTOs to provide information about the project and consumer participants nominated potential carers. Acknowledging the power differential and need to promote consumer autonomy regarding consent, S. D. engaged with consumers in person to explain the ethics process and their choice regarding participation, without adverse consequences for their relationship with the services. Throughout the process, reconsent was sought for observations (verbal) and interviews (signed). As the first author worked as an occupational therapist at the site, consumers known to S. D. and staff members for whom S. D. had supervisory relationships were not recruited to avoid the risk of coerced participation.

Ethnographic observations of care planning discussions among clinicians and between clinicians, consumers and their carers were conducted over 6 months by S. D. This was inclusive of a range of settings including clinical review meetings attended by clinicians, outpatient doctor's appointments and informal conversations between clinicians. More than 44 observations of different settings were conducted and recorded, which included observations of multiple combinations of members from the multidisciplinary teams. Detailed records of observations were kept, including noting of context, participants' present, speech acts and postures and use of a low‐inference vocabulary, to present facts without judgement or interpretation. Reflective notes were kept by S. D. that commented on perceived impacts on those present, including dominant voices and silences. Following this, 12 months of further observations and interviews related to eight consumer participants' care journeys were undertaken. The interview schedule was informed by the early data (from the observations) and feedback on this from two focus groups with clinicians working at the site, and consultation with two individuals with lived experience of mental illness. The themes explored in the interviews included insight, capacity, risk, SDM, engagement and trauma.

Carspecken^1^ provided a rigorous framework of analysis that was used alongside data collection. Data analysis involved low‐level coding, initial meaning reconstructions and validity horizon analysis to identify unarticulated cultural themes. Validity horizon analysis involved two stages. First, the researchers articulated truth claims from the data that were objective (claims accepted by all participants), subjective (likely individual claims) and normative (claims that indicated backgrounded cultural assumptions). Second, the researchers identified which of the claims were foregrounded or backgrounded by participants. Higher level coding was undertaken after several validity horizon analyses were articulated and involved increased abstraction based upon these analyses. QSR NVivo 12 software was used to categorize codes. See Supplementary Appendix for an example of data analysis. Reflexive methods were used throughout the study, including use of a fieldwork journal, regular peer debriefing with the research team (one of whom has lived experience as a consumer and carer) and conducting member checks (through focus groups with clinicians and advisors with lived experience of mental illness). The final stages of Carspecken's^1^ research methodology involved situating the findings within sociological theories to provide in‐depth and critical understandings of the broader (e.g., political) influences on the culture of care planning at the study site.

## 3 FINDINGS

In total, over 40 h of observations of care planning discussions and 37 interviews with consumers, carers and clinicians were conducted. At the outset of data collection, the total number of consumers registered at the study site was 1130, with 92 consumers on a CTO. The first stage of observations allowed for the opportunity to observe different combinations of clinicians working at the site in various groupings, and consumers attending for treatment. Clinical team members (*n *= 75) included medical staff, occupational therapists, mental health nurses, social workers and psychologists. Table 1 presents the demographic details of the participants who engaged in the second stage of the research project: the consumer care journeys.

**Table 1:** Demographics of care journey participants

Participant	Numbers	Demographic details
Consumers		
	8	One woman and 7 men, age 19–49 years (median = 40). Contact with mental health services: ranging from 5 to 20 or more years. All individuals had been on multiple CTOs (3–8), with many implemented consecutively. Four consumer participants had one hospital admission, one participant had two admissions and one participants had three admissions during data collection. Two consumers were linked to a forensic team during fieldwork. One consumer lived with their family, one with their partner and five consumers lived alone (with four receiving a moderate to high level of psychosocial support) and one participant resided in a community rehabilitation unit. One participant was incarcerated in prison at the conclusion of the study.
Clinicians		
	16	Clinicians included medical (*n* = 7), social workers (*n* = 3) and nursing (*n* = 6). These professional groups represented those professions with the highest staff numbers across the teams. Psychologists did take on care coordination and OTs were excluded from interviews due to the lead researcher's supervisory responsibilities. Clinicians' age range: 25–65 years (median = 49). A total of 11 clinicians had worked between 15 and 30 years in mental health care; the junior doctors had 3–6 months' experience.
Carers		
	6	Four consumer participants agreed for their parents to be interviewed (*n* = 6), two consumer participants declined for their family to be contacted and two consumers did not identify having a carer. To provide a broader carer perspective, four additional carers were recruited. All carers reported having regular contact with their relative, with three families having daily contact.

Abbreviation: CTO, community treatment order.

## 4 THE UNDERMINING OF TRUST IN CARE PLANNING RELATIONSHIPS

The findings from this study (summarized in Table 2) illustrate how the positioning of participants (consumers, carers and clinicians) and power discrepancies (between consumers and clinicians) influenced care planning relationships and specifically, the development of mutual trust. CTOs often resulted in clinicians assuming that consumers had lesser capacity than they did, which stifled the opportunity for genuine engagement. Clinicians striving to work collaboratively with consumers had to navigate this bias within a service culture that emphasized a hierarchy of ‘knowing’, with consumers positioned as having lesser knowledge than clinicians. This positioning was influenced by the prioritisation of a narrow interpretation of risk (by services) over broader conceptualisations of stressors (by consumers and their carers). Consumers' positioning, their subsequent lack of power and care that was focused on the service priorities of treatment compliance undermined the development of trusting relationships between clinicians and consumers. Individual workers were constrained by the dominant service culture, which endorsed care that was task‐based, such as ensuring medication compliance or risk assessment, rather than relational therapeutic engagement. This is encapsulated in the following statement by a carer:

There's a trust issue. How can they get to trust this person?… The system in these cases doesn't seem to allow for the very thing that is the problem: the paranoia; and the need for consistency and relationship building … the whole focus is the injection rather than him … the system needs to establish a relationship with Tom other than just administering the needle. (Father—Tom, Interview)

**Table 2:** Undermining of trust in care planning relationships

Theme	Subthemes
Positioning and power	A mismatch of issues and goals
	The silence of risk
	Clinicians leading decision‐making
	Persuasion, leverage and threats
	Minimizing consumers' concerns

The various subthemes that conjointly served to undermine the development of trust in care planning interactions are presented. To reflect the focus on care planning discussions, illustrations are drawn mainly from ethnographic observations of such discussions. All participants are anonymized.

Excerpts taken from a medical appointment and then follow‐up clinical review for the same consumer will be used to illustrate the first three subthemes identified in Table 2. Caleb was a man in his late 40s who had been seen by mental health services for more than 20 years and had been on multiple and consecutive CTOs. The first excerpt (Box 1) is taken from a medical review.

BOX 1 **Caleb—excerpt from medical review**

**Table:** 

Caleb: Speaking of medications, I've got an opinion on this. I've been putting on a lot of weight. Before I was on a depot and they substituted it with a tablet, and I lost weight … I don't want an injection at all. Can't you look at tablets? Psychiatrist: We're too nervous to do it at this stage as we don't know if you'd take it every day. Caleb: Well, they know I take it every day as they come around every morning and watch me take it. Psychiatrist: At some later point we could look at changing to tablets. Caleb: [sighs] Oh, OK then. Psychiatrist: Look, I'll talk to [care coordinator] about switching. We don't want you to get unwell, as when you get unwell you seem to get in trouble with the police. Caleb: Yeah. [sighs]

The second excerpt (Box 2) is taken from a clinical review that occurred the following week. Clinical reviews were attended by mental health clinicians only; thus, decisions made within this context excluded the consumer and family.

BOX 2 **Caleb—excerpt from a clinical review discussion**

**Table:** 

Psychiatrist: I saw [Caleb] last week. He was complaining about the medication and weight gain. We discussed switching medication … Social worker: He's also mentioned the weight gain to me and he's quite fixated on it … [The] GP [is] looking at a physical health assessment. Having another complex client switch meds? I'm worried about this … I'm wondering if it might be better to change medications after court, as he may forget about it.

## 5 A MISMATCH OF ISSUES AND GOALS

In the medical review, Caleb clearly articulated his concerns with the medication. In general, consumers were concerned with issues related to side‐effects from medications and broader life domains, and clinicians with issues related to risks related to illness and treatment. This resulted in the frequent mismatching of goals between consumers and clinicians. Although clinicians often elicited individuals' concerns and hopes through discussion, this did not always translate into support towards consumer‐identified goals. A lack of in‐depth exploration of what was relevant to consumers was frequently observed during medical reviews. This impacted on consumers' experiences of care and, potentially, their engagement with services. The disparity between the consumers' goals and service goals, and the complexity around this, was acknowledged by some clinicians (Box 3).

BOX 3 **Alex—clinical review discussion**

**Table:** 

Social worker: He's insightless around his illness and history … I tell him these are a sleeping man's goals but I'm here if you want to do more, such as go to the gym or find a job. He's only 31. Occupational therapist: How does he occupy himself? Social worker: Gambling, drug taking, hanging out with friends … I realize I'm making a value judgement about how I think money should be spent, but he could spend it on life goals like going travelling. He will state ‘I want to be left alone’. [Clinical review]

Compounding factors in this disparity included differing personal values and a service focus on treating illness and managing risk. Clinicians found it challenging when consumers' values and goals did not align with their well‐being or were not future‐focused. This was particularly evident when people were precontemplative regarding drug use.

## 6 THE SILENCE OF RISK

Although risk informed many clinical decisions regarding CTO use and enforcement, exploration of risk with consumers regarding this was mostly implicit. The lack of transparent dialogue limited opportunities for consumers to understand clinicians' decision‐making regarding medication or rationale for a CTO, with the issue of managing risk reduced to compliance. The silence of, and prioritisation of narrow conceptualisations of risk, and the resulting power imbalance between consumers and clinicians, however, negatively impacted on the establishment of rapport and trust, and meant that consumers were left with the polarized options of either acquiescing or disengaging with the care planning process and services.

## 7 CLINICIANS LEADING DECISION‐MAKING

Systems processes that excluded consumers from discussions where decisions were made about their care further compounded their solitary position (outside the clinical group) and made it challenging, rather than facilitatory, for clinicians and consumers to engage collaboratively. This reinforced the power differential between clinicians and consumers and impacted negatively on the therapeutic relationship and specifically the development of trust. Decisions regarding CTO use and medication were almost always made by clinicians. This power differential was a foregrounded cultural norm. In the first dialogue (Box 1), Caleb had little influence over any decisions regarding his medication, with the psychiatrist deferring the decision to an unknown time in the future and dependent on discussions with other clinicians. In the second dialogue (Box 2), the clinician's needs (workload and stress), and minimizing of Caleb's concerns, appeared to inform the decision‐making process, more than Caleb's preferences.

Consumers on CTOs frequently had very complex needs. Consequently, at times, significant and recent perceived risks made it challenging for clinicians to engage in genuine SDM around medication. The three following excerpts involve another consumer (Mark) and psychiatrist on two occasions 12 months apart and highlight the challenges for all participants. At the first medical review (Box 4), the psychiatrist was supportive of and responsive to Mark's wish to reduce medication:

BOX 4 **Mark—medical review 1**

**Table:** 

Mark: Are we getting the medication reduced today? Psychiatrist: Yes, how do you feel? How is the medication helping you? Mark: Well, I don't know if it's helping. But I feel fine…Psychiatrist: Definitely we can reduce it. Let me try to review the situation.

At the second appointment (Box 5), although the psychiatrist explored Mark's capacity to make decisions about his treatment, the doctor made the final decision. This was the first medical review scheduled following a long admission where Mark had relapsed and been found in an extremely physically compromised state:

BOX 5 **Mark—medical review 2**

**Table:** 

Psychiatrist: Good to see you. How are you? [There are pauses for each question with Mark giving brief answers.] The issue for me is, after being through the trauma, we need to continue the treatment until you're well. We need to look at the pros and cons. This time I will be a bit firmer. What is your view? Mark: I'm not too keen on it. Psychiatrist: Do you think you need treatment? Mark: No. Psychiatrist: What might happen if you stop? Mark: I don't know. I probably need to be monitored a little bit, to see I don't keep to myself too much… Psychiatrist: So, you think by being active people will see you are ok? You don't need medication? Mark: No, I don't think so. Psychiatrist: So, our plan is organizing for fortnightly depot… We can change it to three‐monthly in the future.

In this instance, the psychiatrist was making a reasonable prediction of an unwelcome event,^13^ (p. 238) based on a significant history of relapses that had been life‐threatening to the consumer. Szmukler and Appelbaum^13^ would not consider this action to be coercive, but rather ensuring that adequate care is provided. Although trusting relationships enabled consumers to express their dissatisfaction with care (Box 4 and 5) and facilitated positive risk‐taking by clinicians working within the system (Box 4) and more transparent discussions in the care planning space (Box 5), the negative consequences, for consumers of the power imbalance in decision‐making, even when necessary, were evident. Mark described the loss of active involvement in decisions regarding his care as ‘disorientating’ (Box 6):

BOX 6 **Mark—research interview**

**Table:** 

Mark: Taking some of the decision‐making away from me is a bit disorientating. I don't like it that much … Researcher: What kind of decisions do you feel involved in? Mark: I can't think of any at the moment … [laughs] … In some cases, yes. But they go their merry old way in some ways … Researcher: So, do you think you have much choice in relation to your care? Mark: Not with the medication, you don't. I'd like to have less medication, but they don't seem to like that idea.

BOX 7 **John—medical review**

**Table:** 

Registrar: OK, I think we'll give the current medication about a month. John: I've always found I'm best on no medications. Registrar: Well, I guess we'll try this first. I guess you're on an order.

BOX 8 **Clinician focus group**

**Table:** 

Doctor: So, she has good engagement with [the nurses] and very poor engagement with me. That's something that I'll often construct. Nurse: Good cop, bad cop. Doctor: I'll say, ‘This is my decision, not the nurses', they just follow my orders’.

It was challenging for clinicians engaging with consumers when there were disparate views, particularly around medication. Mark's understanding of what the clinicians thought about his situation differed from his own interpretation; *They believe that I wasn't looking after myself properly while I was at home, I think. I thought I was doing alright, but they have other ideas*. This challenge is explored further below with illustrations, highlighting the potential for clinicians to resort to coercion in this space.

## 8 PERSUASION, LEVERAGE AND THREATS

Within the context of CTOs, use of persuasion, leverage and threats was apparent in discussions among clinicians and between clinicians and consumers. Paternalistic views, an emphasis on risk mitigation and the positioning of consumers as ‘other’ likely informed this approach. Clinicians were generally explicit in their use of persuasion or leverage, with offers of food vouchers or other supports used for leverage to promote engagement in care. The use of threats, however, was both explicit and implicit, with clinicians not always aware that they were resorting to this means of coercion. The use of threats typically appeared to be a result of worker frustration and a shortcut to achieve compliance from consumers. In the excerpt below, the doctor had provided significant information and choice regarding medications, but in the end resorted to a low‐level threat, reminding John that he was on a CTO and thus needed to comply with the recommendation regarding medication (Box 7).

Use of threats de‐emphasized the value of the therapeutic relationship and was a shortcut to the skilled negotiations that would be required if the person were not on an order. Effectively, there was no opportunity for the consumer to not take medication. Although clinicians were often aware of the impact that coercion (e.g., forcing medication) had on their relationship with the person, the potential pervasiveness of the damage that was being done to the consumer's likelihood to trust clinicians and services was unarticulated, or culturally backgrounded. To illustrate this cultural assumption, in the excerpt below, the doctor believed that the consumer would trust ‘others’ in the service who were not directly involved in implementing the CTO (Box 8).

Occasionally, clinicians and families did not correct an individual's misunderstanding of their CTO status, which Szmukler and Appelbaum^13^ label as deception: *His parents are propagating that he is still on a CTO though he hasn't been on one for years*. (Doctor, Clinical review)

## 9 MINIMIZING CONSUMERS' CONCERNS

Consumers' concerns regarding their care experiences were often minimized by clinicians. When consumers communicated their preferences regarding medications, this typically did not result in changes, with clinicians' approaches to such discussions often paternalistic and disempowering. This contributed to adversarial relationships, with consumers labelled and positioned as noncompliant with treatment if they chose to stop or self‐reduce medication and is an example of consumers being presented with no choice other than to comply. The clinician's comment that Caleb was ‘fixated’ with his weight minimized his experience of side effects and invalidated his concern. The impact that being on a CTO itself had on consumers was also often minimized by clinicians, who viewed the CTO as being in the background of an individual's experience. Additionally, while some consumers considered the CTO benign, others felt strongly that being on a CTO was a negative experience and not about care, but rather about being monitored or under surveillance by the team, which may implicitly feel disempowering:

[The CTO] sort of demoralizes you in some aspects because it takes away your choices, your decision‐making in some respects. [pause] Its compulsory medication which is not always the right thing I don't believe. (Mark, Interview)

I can't get rid of it. It's a heinous thing, it's more for you guys. (Medical review—David)

I think [the CTO] reassures them … It's just like a hands‐on, lets them know where I'm at, what I'm doing. There's no spy, but it gets close. You know what I mean? (Caleb, Interview)

## 10 DISCUSSION

In this study, various factors combined to undermine the development of trust and therefore the usefulness of care planning relationships in supporting recovery. The impact of the obscuring of trust in the care planning context was significant for all participants in the care planning relationship, with the power differential between clinicians and consumers, and service structures (such as consumers' absence from clinical review meetings) creating clear barriers to the development of mutual trust. Trust is considered an enabling factor for social inclusion and personal agency^11^; thus, actions (even unintended) that precluded, rather than promoted, trust during clinical interactions had potentially profoundly negative impacts for consumers beyond the clinical encounter. In a recent study, it was found that consumers' experiences of repeated minor coercive events during care contacts, such as minimizing their negative accounts of their experiences of medications, resulted in the person mistrusting his or her own experience and capabilities.^37^ Major coercive events, such as forced medication, were experienced by consumers as ‘violation and abuse’.^37^ (p. 150)

In this study, many consumers' care experiences were minimized, including the experience of coercion resulting from being on a CTO and the side effects of medication, issues that have been identified as barriers to consumer trust of clinicians.^10^ Conversely, consumers being untrusted as a knower of their own experience had a negative impact on clinicians' views of and engagement with them during care planning discussions, encouraging paternalistic or conflictual dynamics. Clinicians' low assumptions regarding consumers' self‐knowledge may reduce the value that is given to an individual's self‐identified goals, including to reduce, change or cease medication. Cath Roper, an Australian consumer academic, highlights that in the context of forced care, a person is ‘judged incompetent, owing to a lack of insight’ and subsequently dismissed as a person without equal status to clinicians.^38^ (p. 420) Similarly, McMillan et al.^21^ found that clinicians did not fully trust consumers to understand their predicament and need for treatment, and thus pathologized any resistance that they expressed about the CTO and treatment.

Theories of trust that emphasize the importance of services and systems as the gateway to developing trust in clinicians^20^ highlight the importance of recognizing harms that the person may have experienced before their contact with services and harms that current service contacts may be causing. [Correction added on 26 August 2021, after first online publication: Correction on theories of trust in the precedent sentence.] As Vassilev and Pilgrim^22^ (p. 355) caution, consumers may be ‘re‐experiencing betrayed trust’ through service contacts. Clinicians' agendas, which related to psychiatric treatment, often overrode consumers' agendas during care discussions. Additionally, some clinicians resorted to threats to increase compliance and minimize certain perceived risks. Although clinicians acknowledged the impact of coercive practices on the therapeutic alliance, the broader impacts on the person and care planning were unspoken. CTOs appeared to result in clinicians' prejudiced bias (not acknowledging people as knowers of their experience) as well as coercive engagement rather than skilled relational work. Significantly, for consumers, coercive actions were a form of discrimination.

The complexity and nuance of consumers' needs on CTOs need to be considered. The balancing of risk in decision‐making was evident in care planning interactions, although the power imbalance meant that the clinicians' determination of cost versus benefits regarding risk usually dominated. Acknowledging the potential impact of decisional conflict is important as research indicates that low decisional quality can negatively impact on a person's quality of life, experience of treatment and recovery.^39^, ^40^ While Caleb and Mark were both able to express their dissatisfaction with treatment, lack of trust precluded risk‐taking (changing medication) and transparent discussions during care planning (as with Caleb), while trust promoted increased collaboration (as with Mark). Trust, therefore, is a potential solution to problems of risk and the basis for risk‐taking and cooperation,^41^, ^42^ both of which are essential components of effective care planning relationships. The interpretation of risk (what or how much) influences individuals' decision‐making to trust others or services.^20^ The importance of reciprocity in trust is thus very relevant in this context.^12^ In practice, however, the use of low‐level threats by clinicians to ensure that they could trust consumers, and thereby mitigate risk, created a dynamic of mistrust in addition to emphasizing the power differential between participants. This space requires skilled and transparent communication from clinicians.

The study findings highlight the interconnectedness between interpersonal and systems trust, with care planning relationships embedded in the service system.^22^ Given many consumers' previous negative experiences with care and broader systems, clinicians need to work hard to develop trust and demonstrate that they are trustworthy. This does not mean avoiding discussions about concerns of harm, but requires finding transparent ways of exploring risks with consumers and their families, including broadening their understandings of adversities that individuals face,^43^ or reframing risk as needs or issues related to the person's safety.^44^ Consumers have highlighted for decades that, to work in partnership, it is necessary that they are included in care planning meetings.^15^ Additionally, the policy has long stated the need and benefits of including consumers and carers in decision‐making.^3^ Active inclusion in this study, however, remained limited to certain settings and was thereby dependent on the allocated clinicians, rather than facilitated at a systems level. Changing clinical review formats to include consumers and carers as the norm would immediately begin to address their absence in care planning.

For a genuine change, clinicians need to critically reflect on the impact of not trusting individuals as knowers. Fricker's^45^ conceptualisation of ‘testimonial injustice’ (the prejudicial silencing of a minority group) and the means to transform this, through the acknowledgement of this process and cultivation of ‘testimonial justice’ (reflexive awareness and motivation to overcome prejudice), provides a means to counter such discrimination. This critique needs to be incorporated into university and service‐facilitated training programmes for clinicians, with individuals with lived experience playing a key role in the development and provision of such training. A related approach that has been proposed to challenge and reform mental health care services is that of reparative truth and reconciliation.^46^ These are complex processes that bring together survivors of services and mental health clinicians, and ‘aim at forging newly respectful relations and restitution for [psychiatric] harm and wrongdoing’.^46^ (p. 84) While Spandler and Mckeown^46^ acknowledge that some critics believe that broader social action is required for genuine change, the authors promote the above approach as being complementary to positive structural change.

Additionally, evidence‐based tools and approaches can be drawn upon to support such change. Possible strategies include use of decision‐making tools,^47^, ^48^ supporting clinicians to reflect on decision‐making processes,^49^ techniques to link the individual's personal goals to medication use^47^ and approaches to repair ruptures in the therapeutic relationship.^50^ Development of trust at the relational level may facilitate the development of trust in the systems, among consumers^41^; however, clinicians need to demonstrate that they are worthy of such trust. This is especially important in public mental health care, where there is often a lack of continuity in care relationships. It is important to highlight that although this study explored care planning with individuals on CTOs, given the experiences of coercion that are consistently reported by consumers during contacts with mental health care services, regardless of legal status, the study findings and recommendations are considered relevant to all individuals seeking care and treatment from public mental health services.

## 11 CONCLUSION

The findings from this ethnographic study identified that trust was a backgrounded barrier to engagement and was infrequently referenced or articulated during care discussions among clinicians and consumers on CTOs. Although the consequences of coercive practices that were compounding discrimination were often unintended, the findings highlight that for care planning to be meaningful, to promote recovery and avoid causing further harm, the profundity of these consequences needs to be acknowledged and addressed by clinicians and services. Fricker's^45^ conceptualisation of prejudicial ethical injustice and the need to counter this through testimonial justice combined with tools and approaches that support genuine SDM provides a viable means for change.

## 12 CONFLICT OF INTEREST

The authors declare that there are no conflict of interests.

## AUTHOR CONTRIBUTIONS

*Conceptualisation*: Suzanne Dawson, Eimear Muir‐Cochrane, Sharon Lawn, Alan Simpson, *Methodology*: Suzanne Dawson, Eimear Muir‐Cochrane, Sharon Lawn. *Data collection*: Suzanne Dawson. *Data analysis*: Suzanne Dawson, Eimear Muir‐Cochrane, Sharon Lawn. *Writing original draft*: Suzanne Dawson. *Review and editing*: Suzanne Dawson, Eimear Muir‐Cochrane, Sharon Lawn, Alan Simpson.

## Supporting information

Supporting information

## Data Availability

The authors confirm that the data supporting the findings of this study are available within the article and/or its Supporting Information Materials.
