# Supplementary material for: Community treatment orders and care planning: How is engagement and decision‐making enacted?
Source: Health Expect. 2021 Aug 12;24(5):1859–67. doi: 10.1111/hex.13329 (PMC8483198; doi:10.1111/hex.13329)
Supplement: Supplementary file 1 — Supporting information [file HEX-24-1859-s001.docx]

Appendix 1.

**Example of early data analysis**

| **TRANSCRIPT** | **LOW-LEVEL CODING** |
| --- | --- |
| **Excerpt 1**  Jim: I’m looking to getting my drivers licence back. There’s people on the bus and I like that. I don’t like being alone.  Doctor: Do you take your phone on the bus?  Jim: Yep.  Doctor: So driving is boring, you can’t look at your phone while you’re driving.  [Observer Comment: It seems that the doctor is minimising the problem Jim is raising about his licence being suspended, though it is done with humour and rapport is maintained, it could be viewed as patronising or making light of/or the best of a situation that Jim can do nothing about at present.]  **Excerpt 2**  Doctor: How’s your mood been?  Jim: I’m happy if I’m around people, and sad if I’m on my own. The fact that I have to take medication is a bit saddening too, to be honest. I just hope I don’t get any side effects.  Doctor: I’m putting a lot of faith in you putting you on orals.  Jim: The sodium valproate is a bit high.  Doctor: I need you to get a blood test.  Jim: There won’t be any trace in my blood as I haven’t taken it for some time.  Doctor: You need to restart it.  [Initial Meaning Reconstruction: I’m not expecting you to be compliant with medication. I’m giving you a chance and you need to take it up.]  [Observer Comment: The doctor was smiling and easygoing when he said this to Jim. It seemed that engagement was superficial and that this was a missed opportunity to engage further in discussion about pros and cons of medication.] | Consumer goal  Minimising/patronising/  doctor as the expert  Consumer experience/side effects  Minimising consumer experience, expectation to follow advice/ lack of trust  Honesty/transparency  Lack of shared decision-making |

**Example of validity horizon analysis**

| **POSSIBLE OBJECTIVE CLAIMS** | **POSSIBLE SUBJECTIVE CLAIMS** | **POSSIBLE NORMATIVE-EVALUATIVE CLAIMS** |
| --- | --- | --- |
| **Most foregrounded**  Nicola is not likely to continue taking medication.  Non-compliance and drug taking will result in a relapse/deterioration in her mental state.  Renewal of the CTO is required to ensure treatment. | **Most foregrounded**  I’m fed up. The doctor and I attempted to engage but it is not leading to treatment adherence.  I’ve exhausted all options around engagement with services.  We need to offer more assertive case management.  We either take responsibility and control, and apply for a CTO, or take a laissez-faire approach and give back control to Nicola. | **Most foregrounded**  She’s making the wrong/poor choices. |
| **Less foregrounded**  Drug use is driving the problems.  A more coercive stance needs to be taken. | **Less foregrounded**  She’s not taking any responsibility and she is going to stop taking medication.  Nicola should engage in what has been offered by mental health services (NGO supports, therapy groups).  I have attempted to work with Nicola by changing her depot to orals while she is on a CTO and prescribing in dialogue with her. | **Less foregrounded**  The service/system needs to be flexible (doctor).  Care coordination needs to be more assertive (doctor).  Everything that could be done has been done (care coordinator). |
| **Background/remote**  There is poor engagement with mental health services. | **Background/remote**  We need to more assertively engage with Nicola (doctor).  Drug-driven behaviour is frustrating and not what we should be focused on.  The consumer has a choice to engage and improve her life. | **Background/remote**  The expectation is that consumers engage with services: Consumers need to engage with our services.  She’s being a ‘bad’ patient. |
